# Multimodality Imaging Evaluation to Detect Subtle Right Ventricular Involvement in Patients with Acute Myocarditis and Preserved Left Ventricular Ejection Fraction

**DOI:** 10.3390/jcm12134308

**Published:** 2023-06-27

**Authors:** Michela Bonanni, Gianmarco Angelini, Laura Anna Leo, Susanne Anna Schlossbauer, Luca Bergamaschi, Antonio Landi, Giuseppe Massimo Sangiorgi, Cinzia Forleo, Elena Pasotti, Giovanni Pedrazzini, Marco Valgimigli, Francesco F. Faletra, Marco Guglielmo, Anna Giulia Pavon

**Affiliations:** 1 Division of Cardiology, Cardiocentro Ticino Institute, Ente Ospedaliero Cantonale, Via Tesserete, 48, 6900 Lugano, Switzerland; 2 Department of Biomedicine and Prevention, University of Rome “Tor Vergata”, 00133 Rome, Italy; 3 Cardiology Unit, Department of Emergency and Organ Transplantation, Policlinico of Bari, University Hospital, University of Bari Aldo Moro, 70124 Bari, Italy; 4 Unit of Cardiology, IRCCS Policlinico St. Orsola-Malpighi, Department of Experimental, Diagnostic and Specialty Medicine-DIMES, University of Bologna, 40138 Bologna, Italy; 5 Department of Cardiology, Division of Heart and Lungs, Utrecht University Medical Center, Utrecht University, 3584 CX Utrecht, The Netherlands

**Keywords:** cardiovascular magnetic resonance, right ventricle, acute myocarditis feature tracking CMR

## Abstract

**Background:** Evaluation of the right ventricle (RV) in patients with acute myocarditis (MY) remains challenging with both 2D transthoracic echocardiography (TTE) and cardiovascular magnetic resonance (CMR). We examined the incremental diagnostic value of CMR feature tracking (FT) to evaluate RV involvement in patients with myocarditis. **Methods:** We enrolled 54 patients with myocarditis and preserved left ventricle (LV) ejection fraction (EF). The CMR protocol included T2-weighted images for edema detection and late gadolinium enhancement (LGE) images. Global longitudinal strain (GLS) of the left ventricle (LV) and RV free wall strain (CMR-FWS) were obtained with CMR-FT. We identified 34 patients (62%) with inferior and lateral segment (IL-MY) involvement and 20 (38%) noIL-MY in case of any other myocardial segment involved. Here, 20 individuals who underwent CMR for suspected cardiac disease, which was not confirmed thereafter, were considered as the control population. **Results:** TTE and CMR showed normal RV function in all patients without visible RV involvement at the LGE or T2-weighted sequences. At CMR, LV-GLS values were significantly lower in patients with MY compared to the control group (median −19.0% vs. −21.0%, *p* = 0.029). Overall, CMR RV-FWS was no different between MY patients and controls (median −21.2% vs. −23.2 %, *p* = 0.201) while a significant difference was found between RV FWS in IL-MY and noIL-MY (median −18.17% vs. −24.2%, *p* = 0.004). **Conclusions:** CMR-FT has the potential to unravel subclinical RV involvement in patients with acute myocarditis, specifically in those with inferior and lateral injuries that exhibit lower RV-FWS values. In this setting, RV deformation analysis at CMR may be effectively implemented for a comprehensive functional assessment.

## 1. Introduction

Acute Myocarditis (MY) is an inflammatory disease of the myocardium whose diagnosis may be challenging in a sizeable proportion of patients due to the frequent heterogeneity of symptoms, ECG, and echocardiographic findings [1]. Recently, it has been demonstrated that RV involvement in myocarditis has an independent and incremental prognostic value beyond clinical variables and imaging criteria [2]. The “gold standard” for its definitive diagnosis is an endomyocardial biopsy (EMB), which is restricted to selected cases given the procedural risks and the low diagnostic sensitivity in focal diseases [1,3]. Within this framework, the role of multimodality imaging has recently become of increasing importance in the diagnosis of myocarditis [4]. 2D-transthoracic echocardiography (TTE) is usually the first imaging examination performed in the setting of myocarditis; however, cardiovascular magnetic resonance (CMR) is nowadays the reference non-invasive diagnostic tool in suspected myocarditis due to its ability to characterize inflammatory myocardial tissue changes in vivo [4,5]. However, both the “Lake Louise Criteria” (LLC) and more recently the updated LLC [6] for CMR-based diagnosis of myocarditis may still be applicable in selected cases [6,7]. The evaluation of right ventricle (RV) involvement may be challenging [8], even if it has recently been found to represent an independent predictor of a worse prognosis in patients with myocarditis [8]. In 2D TTE, RV function can be expressed in global systolic parameters such as Fractional Area Change (FAC) or tricuspid annular plane systolic excursion (TAPSE) [9,10]. However, subtle involvement of the RV during myocarditis may not be easily captured due to anatomical reasons, RV geometry, and the patient’s acoustic window. Among patients with myocarditis, RV involvement may also be challenging by CMR since thin wall thickness makes the assessment of myocardial edema in T2-weighted images or gadolinium contrast uptake in LGE sequences demanding [8,11]. Myocardial strain parameters represent new quantitative indices of cardiac deformation and are thought to be more sensitive markers of contractile dysfunction than LVEF [12,13]. In particular, CMR feature tracking (FT) is a developing contrast-free quantitative method that uses cine images to evaluate systolic and diastolic myocardial deformation parameters, which have been shown to provide independent and incremental prognostic value over clinical features, LVEF, and LGE in patients with myocarditis [14,15]. However, CMR-FT has never been used to evaluate RV involvement in acute myocarditis. Therefore, the aim of the present study was to investigate the role of CMR-FT in detecting the subtle involvement of the RV in a cohort of patients with myocarditis. 

## 2. Methods

### 2.1. Study Population

All consecutive patients with myocarditis referred to our center between January 2014 and October 2021 who underwent CMR were retrospectively considered for the study. Inclusion criteria were: (1) new ECG abnormalities; (2) a raise in myocardial cytolysis markers (high sensibility troponin T > 14 ng/L); (3) the absence of coronary artery disease; and (4) normal LVEF assessed by 2D TTE. Exclusion criteria included: (1) the presence of acute ischemic heart disease; (2) previous myocarditis; (3) the inability to undergo CMR; and (4) poor cine image quality caused by respiratory motion and arrhythmias during CMR. Acute ischemic heart disease was diagnosed in the presence of severe coronary stenosis detected in the invasive coronary angiography, defined as the presence of a luminal reduction of ≥50% in at least one angiographic view of all epicardial vessels with a diameter exceeding ≥1.5 mm.

A definite diagnosis of acute myocarditis was made according to the modified Lake-Louis criteria as the combined presence of a T1 (presence of mid-wall or subepicardial LGE or increased T1 mapping), higher extracellular volume (ECV), and a T2 criteria, hyperintensity in T2-weighted short-tau inversion recovery (TwSTIR) or increased T2 mapping values [16]. The absence of significant coronary artery disease was excluded by invasive coronary angiography or by coronary computed tomography angiography, where appropriate.

A comparative age and sex match of 20 asymptomatic patients were considered as the control group. All patients were free from known cardiovascular risk factors and underwent CMR for a different suspected cardiac condition, but finally, all cases presented normal CMR findings.

### 2.2. Transthoracic Echocardiography

All patients underwent 2D TTE, which was performed on the day of their hospital admission. Echocardiographic images were obtained with standard transducer positions in the parasternal long-axis, short-axis, and apical 4-, 2-, and 3-chamber views (Epiq 7, Philips, Philips Medical System, Best, The Netherlands), according to standard technique [9]. 

Examinations were analyzed offline on dedicated workstations (QLab Cardiac Analysis ver. 10) by two experienced but independent readers blinded to clinical data (MB, GA). Left ventricular (LV) dimensions, volumes, EF, and global longitudinal strain (GLS) were measured as described in the literature [9]. Normal LV EF was defined according to current guidelines as >55% [10]. RV function was evaluated with Tricuspid Annular Plane Systolic Excursion (TAPSE) and Fractional Area Changes (FAC). Tricuspid pulsed wave Tissue Doppler Imaging (TDI) S’ velocity was obtained by aligning the pulsed wave Doppler sample volume with the basal RV free wall at the tricuspid annulus. RV systolic dysfunction was defined in the presence of FAC < 35%, TAPSE < 17 mm, and s’TDI < 10 cm/s according to the recent guidelines [9]. 

2D TTE speckle LV tracking analyses were performed offline using commercially available software (QLab Cardiac Analysis ver. 10, Philips Healthcare Inc., Best, The Netherlands). In detail, 2D grayscale images of all apical views (apical 4-chamber, 3-chamber, and 2-chamber views) with optimized focus on the LV and frame rate > 50 frames/second were considered for the analysis. The software semi-automatically detected the LV end-diastolic and LV end-systolic myocardial contours, which were thereafter manually corrected in case of discrepancy. The cardiac strain was displayed in a 17-segment model according to AHA guidelines. RV strain was evaluated in an apical 4-chamber view and acquired and analyzed with the same characteristics. Since it is known that the GLS of the septum is mainly affected by LV deformation, it was not considered in the analysis. Therefore, only the FWS values were considered for RV strain. 

### 2.3. CMR Acquisition Protocol

All patients underwent ECG-gated CMR performed using a 3 Tesla unit (Siemens Healthcare, MAGNETOM Skyra, Erlangen, Germany) with a 32-channel phased-array surface receiver coil within their hospital stay. Cine images were acquired using a breath-hold steady-state free precession (SSFP) pulse sequence in long-axis (2-chamber, 3-chamber, and 4-chamber) and short-axis views (8 mm slices, 2 mm gap, 10–15 slices). The presence of myocardial edema was evaluated in T2wSTIR. Short-axis image positions used for T2-weighted images were at the same image positions as the cine images in SSFP, ensuring complete LV coverage. Finally, the myocardial scar was evaluated in late gadolinium enhancement (LGE) images after 0.2 mmol/kg body weight gadobutrol (Gadovist^®^, Bayer Healthcare, Berlin, Germany) and a 10–15 min waiting time. LGE images were acquired using a 2D breath-hold phase-sensitive segmented inversion-recovery Gradient echo (GRE)-based pulse sequences in the same orientations as the cine acquisitions. The inversion time was individually optimized to nullify normal myocardium. 

### 2.4. CMR Analysis

All CMR examinations were analyzed using the Argus software (Siemens Healthiners, Erlangen, Germany) to calculate LV and RV volumes, LV mass, LVEF, and RVEF by delineating endocardial and epicardial borders in the stack of short-axis cine images. The presence of fibrosis by LGE was visually assessed and categorized in epicardial, mid wall, or diffuse/transmural patterns, and its extent was reported according to the AHA 17-segment model [17]. Edema was analyzed using the signal intensity ratio of the myocardium versus skeletal muscle on T2-weighted images [18]. For the assessment of regional T2-weighted, the AHA 17-segment model was also used [17].

CMR-FT analyses were performed using commercially available software (QStrain, Medis Medical Imaging System version 2.0.12.2, Leiden, The Netherlands). Long-axis SSFP cine images were used to derive global longitudinal strain (CMR-GLS). Endocardial and epicardial contours were manually traced from SSFP cine images in the end-diastolic phase. All contours were automatically propagated throughout the cardiac cycle and manually corrected in cases of errors. RV myocardial strain was measured blinded to clinical data under the supervision of an experienced CMR cardiologist (MB, GA supervision, AGP). RV endocardial contours were drawn manually during the end-diastolic and end-systolic phases. Subsequently, the software traced the cardiac contours during all the cardiac cycles, resulting in the peak global longitudinal strain (CMR-GLS) of the entire right ventricle, the GLS of the RV free wall (CMR-FWS), and the GLS of the septum. Similarly to echocardiographic parameters, since it is known that the GLS of the septum is mainly affected by LV deformation, it was not considered in the analysis, so for the RV strain only the CMR-FWS values were considered. 

The reproducibility of 2D speckle tracking analysis and CMR-FT analysis was assessed on a random sample of 20 patients, analyzed by 2 different operators blinded to previous analysis (Supplementary Materials). 

### 2.5. Statistical Analysis

Continuous variables underwent the Shapiro–Wilks test for normality. Continuous variables were expressed as mean ± SD in the case of a normal distribution or as median [interquartile range] in the case of a non-normal distribution. Comparison of continuous variables between two groups was performed using the independent-sample Student’s *t*-test in the case of a normal distribution or the Mann–Whitney test in the case of non-normal distribution. One-way analysis of variance (ANOVA) was used to determine statistically significant differences in the presence of three or more independent groups. A Bonferroni correction for post-hoc analysis was used. 

The association between any categorical variable was analyzed using Fisher’s exact test or the chi-square test. Correlation analysis was performed using Spearman’s correlation test. Reproducibility analyses for the measurement of 2D speckle tracking analysis and CMR-FT parameters were performed using Pearson’s correlation and Bland–Altman statistics.

Considering AHA segmentation, we identified 6 macro-areas: anterior wall (AHA 1, 7, 13), anteroseptal wall (AHA 2, 8, 14), anterolateral wall (AHA 6, 12), inferior wall (AHA 4, 10, 15), inferolateral (AHA 5, 11, 16), and inferoseptal wall (AHA 3, 9). The study population was divided into two groups according to myocardial wall involvement. In the IL-MY, group we considered all patients presenting inferior and lateral LV wall abnormalities, while the noIL-MY group was made up of all patients without any abnormalities detected in the inferior and lateral LV myocardial walls. All statistical analyses were performed using SPSS 25.0 (StataCorp, College Station, TX, USA). A *p*-value of <0.05 was considered statistically significant.

## 3. Results

### 3.1. Baseline Characteristic of Population

Among the 60 patients screened for eligibility, 4 patients were excluded since they did not undergo CMR due to claustrophobia. Two additional patients were excluded due to poor imaging quality. Overall, 54 patients with myocarditis were finally included in the study. The baseline and clinical characteristics are presented in Table 1. Patients with myocarditis were younger than controls (36.6 ± 17.3 vs. 55.6 ± 8.8 years old; *p* < 0.001) and predominantly male (92.2% vs. 60%, *p* < 0.001). There was a similar prevalence of cardiovascular risk factors between the two groups. Among patients with myocarditis (Table 2), chest pain was the predominant symptom at clinical presentation (98.1%), whereas fever or flu-like symptoms occurred in 33 patients (61.1%), dyspnea in 31 patients (57.4%), and palpitation in 10 patients (18.5%). ECG abnormalities were found in 41 patients (75.9%), of whom 34 (62.9%) had ST-segment elevation and 10 (18.5%) had ST-segment depression or T-wave inversion. Laboratory testing at admission demonstrated an increase in inflammation markers and cardiac enzymes in all patients with a median hs-TnT value of 1785.5 ng/mL [IQR 742–8319.5]. In total, 50 patients underwent invasive coronary angiography that was negative for significant coronary stenosis, while in 4 patients, coronary artery disease was excluded due to young age (<20 years old). All patients were hemodynamically stable with no cardiac arrhythmias during hospitalization and were discharged free of symptoms.

### 3.2. 2D Transthoracic Echocardiography

All patients underwent 2D TTE on the day of their hospital admission. Patients with myocarditis presented normal LV EF, which was not statistically different from the control group (58.6% [53.2–60.1%] vs. 59.3% [54.1–64.2%], *p* = 0.071). No evident LV and RV wall motion abnormalities were detected (Table 1). Likewise, diastolic filling and LV geometry were normal in all patients, with no evidence of LV hypertrophy and/or dilatation. No significant valvular heart diseases were observed. In patients with myocarditis, mild pericardial effusion was noted in 7 cases (13%). 

Speckle tracking analysis was feasible in all patients with myocarditis and in the control group. In 2D TTE, LV GLS values were significantly lower in patients with myocarditis compared to the control group (−16.4% [−14.2–−20.1%] vs. −22.4% [−18.2–−25.1%], *p*-value = 0.017). 2D TTE findings are summarized in Table 2. 

### 3.3. Cardiovascular Magnetic Resonance Parameters

**Figure 1:**
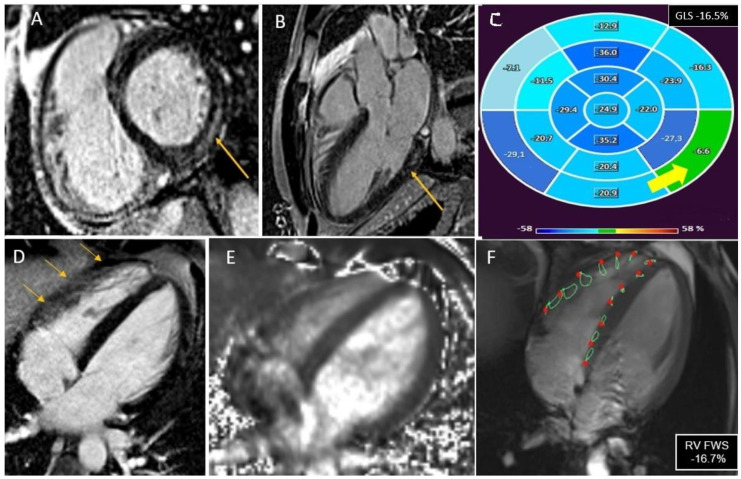
CMR assessment in a 34-year-old patient with IL-MY showing the presence of subepicardial scar in the inferolateral wall that can be seen in LGE sequence in basal short axis view (Panel (**A**), yellow arrow) and 3-chamber view (Panel (**B**), yellow arrow). Patient was also diagnosed with pericarditis involving the diaphragmatic layer of the pericardium (green arrow) with mild pericardial effusion (blue stars). The LV GLS is −16.5%, reduced in comparison to normal values, especially in the presence of myocardial scar (Panel (**C**), yellow arrow). No necrosis/scar was detected in LGE sequences in the RV (Panel (**D**), yellow arrow), and no edema was evident in T2 mapping, despite thin wall thickness limiting the diagnosis (Panel (**E**)). On the contrary, RV FWS was reduced (−16.7%) compared to normal values, highlighting a possible subtle RV involvement (Panel (**F**)).

The mean time from 2D TTE to CMR was 1.9 ± 1.2 days. CMR findings are summarized in Table 2. The totality of patients diagnosed with myocarditis presented myocardial edema and LGE at the CMR evaluation. The distribution of LGE was mainly subepicardial (35 patients, 64.8%) rather than mid-wall (19 patients, 35.1%). The median value of total necrosis burden was 3 (1–5) numbers of segments, and the median value of myocardial edema was 2 (1–6) numbers of segments. The CMR LV-GLS values were significantly lower in patients with myocarditis compared to the control group (−19.0% [−15.2–−27.3%] vs. −21.0% [−18.4–−29.7%], *p* = 0.029). Considering LGE and edema distribution found in CMR, inferior segments were involved in 28 patients (51.8%), infero-lateral segments in 19 patients (35.1%), antero-lateral in 8 patients (14.8%), anterior segments in 11 patients (20.3%), infero-septal in 14 patients (25.9%), and antero-septal segments in 7 patients (12.9%). Overall, 34 patients (62.9%) had myocarditis involving both inferior and lateral segments (IL-MY), whereas 18 patients (33.3%) did not present such involvement (noIL-MY). The baseline characteristics of IL-MY and noIL-MY are summarized in Table 2. In particular, no significant differences were found between IL-MY and noIL-MY concerning CMR LV-GLS values (−17.4% [−14.2–−27.3%] vs. −18.8% [−15.7–−26.8%]; *p* = 0.365) (Figure 1).

### 3.4. Right Ventricle Involvement

All patients presented normal RV function at 2D TTE. In particular, TAPSE and S’ TDI values were found within normal ranges in all patients with myocarditis as well as in control groups (TAPSE: 22.7 ± 2.2 mm vs. 23.9 ± 2.6 mm, *p* = 0.068; s’TDI: 11.2 [9.7–13.1] cm/s vs. 9.9 [8.7–12.9] cm/s, *p* = 0.071) and also in IL-MY and noIL-MY TAPSE was within the normal range (22.5 ± 1.9 mm vs. 23.0 ± 2.8 mm, *p* = 0.460) as well as s’ TDI (10.9 [9.8–12.8] cm/s vs. 9.9 [9.2–12.7] cm/s *p* = 0.125). FAC values were found within the normal range in all patients: patients with myocarditis did not present any statistically significant difference with the control group (41.6 ± 3.8% vs. 42.8 ± 3.5%, *p* = 0.212), likewise, no statistically significant difference was found in IL-MY and noIL-MY patients (41.2 ± 3.9% vs. 42.3 ± 3.8%; *p* = 0.301). 

In CMR examination, the RV volumes were in the normal range (80.1 mL/m^2^ [69.2–92.1 mL/m^2^]) as the RV-EF (67.7 mL/m^2^ [51.3–81.5 mL/m^2^]) and no abnormal wall motion was detected in cine images. Moreover, no signs of RV involvement in terms of edema on T2-weighted images or LGE were found. 

**Figure 2:**
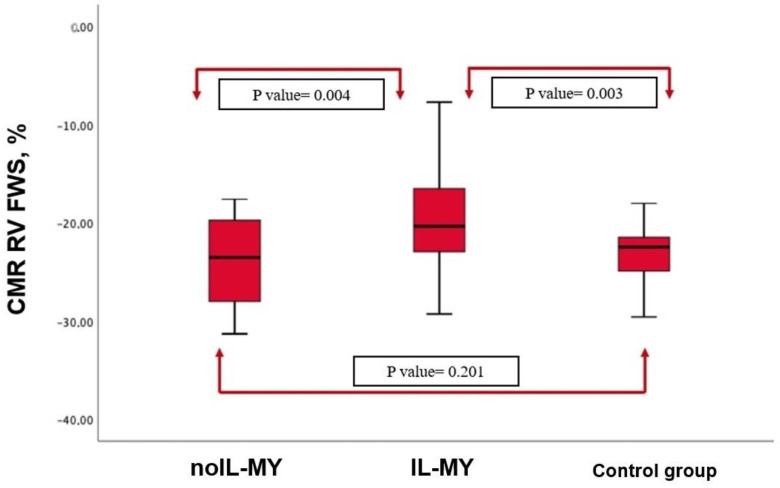
Cardiac Magnetic Resonance (CMR) Right Ventricle (RV) free wall strain (FWS) in patients with MY, in the subgroup of IL-MY, and control group. Data are shown as boxplots.

CMR RV-FWS was no different in patients with myocarditis compared to controls (−21.2% [−17.4–−29.3%] vs. −23.2% [−18.2–−27.3%], *p* = 0.201), while a statistically significant difference was found between RV-FWS in IL-MY and noIL-MY (−18.17% [−11.2–−22.8%] vs. −24.2% [−19.4–−28.7%], *p* = 0.004). (Figure 1 and Figure 2). Finally, considering a median value of RV FWS −23% in the control group, 32 patients (59.2%) with myocarditis were found to have lower values, with a statistically significant difference among IL-MY and noIL-MY (73% vs. 38%, *p* = 0.003). (Figure 2).

## 4. Discussion

This study evaluates the diagnostic value of CMR-FT-derived myocardial strain parameters in detecting RV involvement in patients with myocarditis and preserved EF, specifically differentiating between inferolateral and non-inferolateral acute myocarditis.

The main findings of the study are: (1) myocardial GLS in 2D TTE and CMR-FT is significantly reduced in all patients with myocarditis; (2) in patients with myocarditis and preserved ejection fraction, subtle RV involvement has been found in the presence of large myocardial involvement and acute inferolateral localizations; and (3) CMR-FT is able to detect subtle RV involvement on top of routine markers.

Even if EMB is the gold standard in the diagnosis of myocarditis, it is an invasive procedure and has the inheritable drawback of sampling errors, so cardiovascular imaging is now considered crucial in the evaluation of patients with myocarditis. Previous studies identified 2D speckle-tracking TTE as a useful tool in the diagnostic work-up of myocarditis [14,15] Our analysis is in line with previous findings since, in our population, lower values of 2D TTE GLS are present in cases of myocarditis.

However, due to its unique capacity for tissue characterization, CMR has recently become the preferred imaging modality to evaluate patients with suspected myocarditis. In this setting, CMR-FT is a novel tool that has been shown to have an important diagnostic role in patients with myocarditis by better evaluating LV myocardial deformation [14,15]. In the first report by Luetkens et al. [6], CMR-FT measurements were shown to have good diagnostic accuracy in confirming the presence of myocarditis. Also, Fischer K. et al. recently highlighted that myocardial strain using CMR-FT may serve as a novel marker to improve risk stratification in myocarditis and that GLS of the LV has been shown to provide independent and incremental prognostic value [14,15]. Also in our population, the presence of LV GLS in CMR was found to be lower in patients with myocarditis and normal EF compared to controls, confirming that the presence of LV GLS changes may be useful to identify and confirm the diagnosis of myocarditis [14].

So far, the role of RV involvement in myocarditis has been poorly investigated. Recently, Bernhard et al. evaluated the prognostic role of RV involvement in a large cohort of patients with acute myocarditis. They found that RV ejection fraction at CMR is an independent and incremental predictor for adverse cardiovascular events and strongly suggested including this parameter in future risk stratification. Actually, it is well known that patients with RV involvement present with worse clinical and hemodynamic conditions. Since EMB in the RV is challenging and usually gathered from the right side of the interventricular septum, which may be more reflective of the LV, the actual prevalence of RV myocarditis may be significantly underestimated. Yilmaz et al. performed EMB in 481 patients with clinically suspected myocarditis and impaired LV function, highlighting that biventricular involvement is present in nearly 70% of patients [19]. LV dysfunction and histologically proven myocarditis in the RV were found in 89 of 127 patients undergoing EMB for clinically suspected myocarditis [3]. It is also worth mentioning that the myocarditis involving the RV may mimic arrhythmogenic RV cardiomyopathy (ARVC), as demonstrated by Chimenti et al. [20], who found signs of myocarditis in EMB in 70% of patients with imaging suspicion of ARVC. Finally, active RV myocarditis may evolve into a chronic scar that could be the substrate for ventricular arrhythmias and dysfunction. Aquaro GD et al. [8] demonstrated a prevalence of 17.8% RV involvement at the CMR evaluation in hemodynamically stable patients with myocarditis and preserved LVEF. In the large cohort published by Bernhard et al. [2], among 1125 consecutive patients with suspected myocarditis, signs of RV involvement were present in 25% of cases. They demonstrated the independent and incremental prognostic value of RVEF beyond clinical variables, CMR tissue characterization, and LV function. RV involvement is typically evaluated by detecting myocardial necrosis/scar in LGE or the presence of myocardial edema in T2-weighted images. However, it must be considered that the evaluation of RV involvement in the setting of myocarditis is affected by an extremely thin wall thickness, so edema and scar detection may be very challenging, and the final exact prevalence of the disease can still be underestimated. CMR-FT may overcome this main limitation by evaluating myocardial deformation and has been largely used in the evaluation of LV [10,14]. However, to date, no data on the CMR-FT of the RV have been considered.

In our study, none of the patients with acute myocarditis showed RV involvement according to TTE. RV was found to have normal values in all cases, and no functional alterations were documented according to TAPSE, s’TDI, or FAC evaluation. Similarly, in CMR, RV volumes were in the normal range, and on top of that, T2-weighted images did not show the presence of edema, nor were signs of necrosis detected on LGE. Despite the results being in the normal range according to standard criteria [8,9], in our population, patients with IL-MY have a lower value of RV FWS compared to noIL-MY and control groups. To note, 32 (61%) patients with myocarditis were found to have lower RV FWS values than the median value of RV FWS in the control population, with the majority of patients in the IL-MY group (73% vs. 38%, *p* = 0.003). All these features suggest the possible presence of subtle RV involvement in patients with IL-MY. A possible explanation for that may be linked to the presence of pericardial involvement, which was also more frequent in patients with IL-MY, so RV may be either involved due to direct contiguity with the inferolateral LV wall or as a consequence of adjacent pericardial inflammation. Finally, it is worth mentioning that this subgroup of patients tends to present a larger area of myocardial edema and necrosis detected in T2-weighted images and LGE compared to patients in noIL-MY groups.

In our study, the use of CMR-FT on the RV proved to be an effective tool to identify subtle RV involvement even in the absence of clear RV dysfunction or in the presence of necrosis/scar and edema. In this setting, the value of strain imaging might be of interest as it allows the identification of regional myocardial deformation that may evolve into ventricular dysfunction. Further studies are needed to explore the prognostic role of CMR-FT in detecting RV involvement in myocarditis in patients with preserved but also impaired LVEF.

## 5. Limitations

The present study presents some limitations. First of all, it is a retrospective study with relatively small sample size, and only patients with normal EF were considered. For this reason, no EMB as a reference standard was performed to diagnose myocarditis, which was defined by combining clinical and laboratory data and suggestive CMR findings, as previously reported. In this setting, the sensitivity and specificity of 2D TTE and CMR cannot be tested in this specific population. Even though our study population was young, without significant comorbidities, it must be kept in mind that alteration of myocardial strain parameters is non-specific and can be found in many cardiac diseases, so myocardial strain analysis must always be interpreted within the appropriate clinical context. The role of the 2D TTE RV strain was not assessed in most of the population with myocarditis due to poor acoustic window, which limited the accuracy of tracking. To note, patients with acute myocarditis in our population did not show any impairment of the RV function in both TTE and CMR standard parameters, so the role of CMR-FT in this subcategory of patients needs further evaluation. Finally, due to the retrospective nature of the study, no dedicated clinical and instrumental follow-up was planned at our institution, so the data were available only for 52% of patients. In all these cases, patients were clinically stable, and no RV dysfunction was assessed at TTE. However, due to the number of patients lost at follow-up, the implication of subtle RV impairment needs to be explored in a dedicated prospective study enrolling a larger population. Finally, no patients with RV involvement and preserved LV were admitted to our center during the study period, and not all patients with initially suspected myocarditis later confirmed to be free of the condition were routinely addressed to CMR, so the ability of CMR-FT to evaluate RV myocardial deformation in these specific populations cannot be assessed in the present study.

## 6. Conclusions

In hemodynamically stable patients with normal LV EF with myocarditis, CMR-FT can discriminate between diseased and healthy patients. Strain parameters represent a standardized unit of measurement and can be determined using different imaging techniques (2D-TTE or CMR). Subtle involvement of the RV can be detected by CMR-FT in addition to the standard technique, and it is usually associated with inferolateral or inferior myocarditis.

## Figures and Tables

**Table 1:** Baseline clinical characteristics, Echocardiography, and CMR results of the study population with myocarditis and control group. SD, standard deviation; CAD, coronary artery disease; EDVi, end-diastolic volume index; LV, left ventricle; EF, ejection fraction; 2D, two dimensional; LAVi, left atrial volume index; TAPSE, tricuspid annular plane systolic excursion; FAC, fractional area change; CMR, cardiac magnetic resonance; GLS, global longitudinal strain; RV, right ventricle; FWS, free wall strain; ESVi, end-systolic volume index.

		Myocarditis	Controls	*p* Value
		*n* = 54	*n* = 20	
Clinical Characteristics				
Age (years)	Mean ± SD	36.6 ± 17.3	55.6 ± 8.8	<0.001
Male	*n* (%)	49 (92.2)	12 (60)	<0.001
Body Surface Area (m^2^)	Mean ± SD	1.90 ± 0.16	1.85 ± 0.12	0.110
Dyslipidemia	*n* (%)	9 (17.3)	7 (35)	0.106
Arterial Hypertension	*n* (%)	4 (7.7)	4 (20)	0.137
Diabetes	*n* (%)	2 (3.8)	3 (15)	0.09
Current smoker	*n* (%)	18 (34.6)	10 (50)	0.246
Familiarity	*n* (%)	7 (13.5)	4 (20%)	0.490
History of CAD	*n* (%)	2 (3.8)	2 (10)	0.307
Echocardiography results				
EDVi mL/m^2^	Median [IQR]	60.7 [40.2–79.1]	59.2 [36.1–76.2]	0.645
LV EF 2D %	Median [IQR]	58.6 [53.2–60.1]	59.3 [54.1–64.2]	0.071
LV GLS, %	Median [IQR]	−16.4 [−14.2–−20.1]	−22.4 [−18.2–−25.1]	0.017
LV Mass Index g/m^2^	Median [IQR]	79.1 [61.0–85.4]	69.8 [53.0–79.4]	0.063
E/A	Mean ± SD	1.24 ± 0.75	1.03 ± 0.32	0.327
E/e’	Mean ± SD	6.23 ± 2.96	7.55 ± 2.2	0.076
LAVi mL/m^2^	Mean ± SD	23.8 ± 7.0	22.5 ± 4.9	0.460
TAPSE mm	Mean ± SD	22.7 ± 2.2	23.9 ± 2.6	0.068
FAC %	Mean ± SD	41.6 ± 3.8	42.8 ± 3.2	0.212
CMR results				
LV EF%	Median [IQR]	57.6 [55.2–61.3]	59.3 [54.6–65.3]	0.073
LV GLS %	Median [IQR]	−19.0 [−15.2–−27.3]	−21.0 [−18.4–−29.7]	0.029
RV FWS%	Median [IQR]	−21.2 [−17.4–−29.3]	−23.2 [−18.2–−27.3]	0.201
RV EDVi mL/m^2^	Median [IQR]	80.1 [69.2–92.1]	88.1 [69.5–98.1]	0.076
RV ESVi mL/m^2^	Median [IQR]	27.5 [24.2–37.2]	28.0 [21.2–36.1]	0.231
RV EF%	Median [IQR]	49.6 [47.2–59.3]	48.7 [46.6–57.3]	0.456

**Table 2:** 2D echocardiography and Cardiovascular Magnetic Resonance data on study populations. AM, acute myocarditis; RV, right ventricle; EDVi, end-diastolic volume index; SD, standard deviation; EF, ejection fraction; 2D, two dimensional; LV, left ventricle; LAVi, left atrial volume index; TAPSE, tricuspid annular plane systolic excursion; FAC, fractional area change; CMR, cardiac magnetic resonance; GLS, global longitudinal strain; FWS, free wall strain; ESVi, end-systolic volume index; LGE, late gadolinium enhancement. * *p* values are referring to a comparison between IL-MY and noIL-MY.

		All Myocarditis	IL-MY	noIL-MY	*p* Value *
		*n* = 54	*n* = 34	*n* = 20	
Echocardiography results					
EDVi mL/m^2^	mean ± SD	60.75 ± 15.2	60.47 ± 17.96	66.39 ± 20.84	0.290
EF 2D %	mean ± SD	56.23 ± 7.22	55.32 ± 7.93	57.94 ± 5.44	0.216
LV Mass Index g/m^2^	mean ± SD	79 ± 20	80.82 ± 23.39	75.56 ± 11.35	0.373
E/A	mean ± SD	1.24 ± 0.75	1.24 ± 0.91	1.25 ± 0.34	0.976
E/e’	mean ± SD	6.23 ± 2.96	6.38 ± 3.01	5.94 ± 2.92	0.617
LAVi mL/m^2^	mean ± SD	23.8 ± 7.05	23.6 ± 8.0	24.2 ± 4.8	0.772
TAPSE mm	mean ± SD	22.7 ± 2.2	22.5 ± 1.9	23.0 ± 2.8	0.460
FAC %	mean ± SD	41.6 ± 3.8	41.2 ± 3.9	42.3 ± 3.8	0.301
CMR results					
LV EF%	median (IQR)	58.6 [53.2–60.1]	59.4 [54.2–63.1]	57.3 [55.1–64.2]	0.256
LV GLS %	median (IQR)	−19.0 [−15.2–−27.3]	−17.4 [−14.2–−27.3]	−18.8 [−15.7–−26.8]	0.365
LV segments with edema	median (IQR)	2 [1–6]	3 [2–7]	2 [1–6]	0.015
LV segments with LGE	median (IQR)	3 [1–5]	3 [2–6]	2 [1–6]	0.022
RV EF%	median (IQR)	67.7 [51.3–81.5]	47.1 [45.2–53.4]	48.6 [46.3–58.2]	0.256
RV EDV mL/m^2^	median (IQR)	80.1 [69.2–92.1]	85.1 [76.0–95.4]	79.0 [69.0–91.1]	0.06
RV ESV mL/m^2^	median (IQR)	27.5 [24.2–37.2]	28.2 [23.4–39.1]	31.3 [24.5–39.2]	0.560
RV FWS %	median (IQR)	−21.2 [−17.4–−29.3]	−18.17 [−11.2–−22.8]	−24.2 [−19.4–−28.7]	0.004
RV FWS < −23%	*n* (%)	32 (61%)	25 (73%)	7 (38%)	0.003
Pericardial effusion	*n* (%)	7 (13.5)	5 (14.7)	2 (11.1)	0.718
Pericardial LGE	*n* (%)	14 (27)	11 (32)	3 (17)	0.002

## Data Availability

Data are available to corresponding author under reasonable request.

## Supplementary Materials

The following supporting information can be downloaded at: https://www.mdpi.com/article/10.3390/jcm12134308/s1.

## Abbreviations

MY	Acute myocarditis
CMR	Cardiovascular magnetic resonance
CMR-FT	Feature tracking CMR
EF	Ejection fraction
FAC	Fractional area change
GLS	Global longitudinal strain
LGE	Late gadolinium enhancement
LV	Left ventricle
LV-GLS	Left ventricle global longitudinal strain
RLS	Regional longitudinal strain
RV	Right ventricle
RV FWS	Right ventricle free wall longitudinal strain
TAPSE	Tricuspid annular plane systolic excursion
TTE	Transthoracic echocardiography
