# Supplementary material for: Multimodality Imaging Evaluation to Detect Subtle Right Ventricular Involvement in Patients with Acute Myocarditis and Preserved Left Ventricular Ejection Fraction
Source: J Clin Med. 2023 Jun 27;12(13):4308. doi: 10.3390/jcm12134308 (PMC10342404; doi:10.3390/jcm12134308)
Supplement: Supplementary file 1 [file jcm-12-04308-s001.zip › jcm-2319896-supplementary.pdf]

| | | All Myocarditis | IL-MY | noIL-MY | P value |
|---|---|---|---|---|---|
| | | ***N=54*** | ***N= 34*** | ***N= 20*** | |
| ***Clinical Characteristics*** | | | | | |
| Age (years) | mean ± SD | 36.6±17.3 | 36.2 ±17.2 | 37.22±17.7 | 0.852 |
| Males | n (%) | 49 (92.2) | 32 (92.1) | 17 (94.4) | 0.962 |
| Body Surface Area ($m^2$) | mean ± SD | 1.90±0.16 | 1.94±0.18 | 1.84±0.14 | 0.065 |
| Dyslipidemia | n (%) | 9 (17.3) | 6 (17.6) | 3 (16.7) | 0.929 |
| Arterial Hypertension | n (%) | 4 (7.7) | 3 (8.8) | 1 (5.6) | 0.962 |
| Diabetes | n (%) | 2 (3.8) | 0 (0) | 2 (11.1) | 0.165 |
| Current smoker | n (%) | *18 (34.6)* | 12 (35.3) | 6 (33.3) | 0.570 |
| Familiarity | n (%) | 7 (13.5) | 5 (14.7) | 2(11.1) | 0.718 |
| History of CAD | n (%) | 2 (3.8) | 2 (5.9) | 0 (0) | 0.294 |
| ***Clinical presentation*** | | | | | |
| Chest pain | n (%) | 51 (98.1) | 34 (100) | 17 (94.4) | 0.165 |
| Fever | n (%) | 33 (63.5) | 23 (67.6) | 10 (55.6) | 0.389 |
| Palpitation | n (%) | 10 (19.2) | 5 (14.7) | 5 (27.8) | 0.255 |
| Dyspnea | n (%) | 31 (59.6) | 20 (58.8) | 11 (61.1) | 0.873 |
| ***ECG*** | | | | | |
| ECG abnormalities | n (%) | 41 (78.8) | 27 (79.4) | 14 (77.8) | 0.728 |

| | | | | | |
|---|---|---|---|---|---|
| Negative T-wave | n (%) | 10 (19.2) | 7 (20.6) | 3 (16.7) | 0.523 |
| ST-elevation, | n (%) | 34 (65.4) | 22 (64.7) | 12 (66.7) | 1.0 |
| ***Laboratory testing*** | | | | | |
| Leukocytes ($10^3$ cells/ml), | median (IQR) | 7.1 (3.4-11.8) | 7.1 (2.9-10.7) | 7.9 (4.1-12.3) | 0.451 |
| Troponine I peak ng/ml, | median (IQR) | 1785.5 [742-8319.5] | 1546 [832-8279] | 3850 [426-8777.5] | 0.773 |
| CRP mg/dl, | median (IQR) | 55 [28-73.5] | 55.5 [23.5-76.7] | 54 [34.2-65.5] | 0.571 |

**Table S1 Supplementary Materials:** clinical characteristics of patient with myocarditis.

MY, acute myocarditis; LGE, late gadolinium enhancement; SD, standard deviation; CAD, coronary artery disease; IQR, interquartile range; CRP, C- reactive protein.

**Supplementary Materials: baseline characteristics and clinical indication for CMR of control population**

## Intra- and inter-observer reproducibility of CMR-FT and 2D TTE speckle tracking measurements:

Intra-observer (r=0.85, p<0.001/Mean difference 1.23% −95% levels of agreement— -18.12% to -21.71% ms) and inter-observer (r=0.91, p<0.001/Mean difference 1.78% ms−95% levels of agreement— -19.01 to 23.71%) reproducibility for CMR RV FWS was excellent. Intra-observer (r=0.65, p=0.003/Mean difference 1.23% −95% levels of agreement— -18.12% to -23.42% ms) and inter-observer (r=0.91, p<0.001/Mean difference 1.78% ms−95% levels of agreement— -19.01 to 24.48%) reproducibility for CMR LV GLS was excellent. Intra-observer (r=0.71, p=0.004/Mean difference 2.41% −95% levels of agreement— -16.22% to -24.41% ms) and inter-observer (r=0.84, p=0.005/Mean difference 1.18% ms−95% levels of agreement— -17.14 to 22.42%) reproducibility for 2D TTE LV GLS was good.
